# Surgical Audit of Patients with Ileal Perforations Requiring Ileostomy in a Tertiary Care Hospital in India

**DOI:** 10.1155/2015/351548

**Published:** 2015-07-13

**Authors:** Hemkant Verma, Siddharth Pandey, Kapil Dev Sheoran, Sanjay Marwah

**Affiliations:** Department of Surgery, Pt. B.D. Sharma, PGIMS, Rohtak 124001, India

## Abstract

*Introduction*. Ileal perforation peritonitis is a frequently encountered surgical emergency in the developing countries. The choice of a procedure for source control depends on the patient condition as well as the surgeon preference.* Material and Methods*. This was a prospective observational study including 41 patients presenting with perforation peritonitis due to ileal perforation and managed with ileostomy. Demographic profile and operative findings in terms of number of perforations, site, and size of perforation along with histopathological findings of all the cases were recorded.* Results*. The majority of patients were male. Pain abdomen and fever were the most common presenting complaints. Body mass index of the patients was in the range of 15.4–25.3 while comorbidities were present in 43% cases. Mean duration of preoperative resuscitation was 14.73 + 13.77 hours. Operative findings showed that 78% patients had a single perforation; most perforations were 0.6–1 cm in size and within 15 cm proximal to ileocecal junction. Mesenteric lymphadenopathy was seen in 29.2% patients. On histopathological examination, nonspecific perforations followed by typhoid and tubercular perforations respectively were the most common.* Conclusion*. Patients with ileal perforations are routinely seen in surgical emergencies and their demography, clinical profile, and intraoperative findings may guide the choice of procedure to be performed.

## 1. Introduction

Ileal perforation peritonitis is a frequently encountered surgical emergency in the developing countries [1]. Typhoid is the most common cause for this dreaded complication while tuberculosis, trauma, and nonspecific enteritis follow close suit [2]. The incidence of perforation in typhoid fever has been reported to be 0.8% to 18% [3]. Tuberculosis accounts for 5–9% of all small intestinal perforations in India and it is the second commonest cause after typhoid fever [4]. These cases of perforation peritonitis often require ileostomy as a lifesaving measure. However, in the Western countries, indications for ileostomy are altogether different and include inflammatory bowel disease, familial adenomatous polyposis, colorectal cancer, pelvic sepsis, trauma, diverticulitis, fistula, ischemic bowel disease, radiation enteritis, fecal incontinence, and paraplegia [5].

The standard source control measure for secondary peritonitis due to hollow viscus perforation is resuscitation followed by laparotomy. The methods of source control for ileal perforations include primary closure, resection, and anastomosis of small gut or diverting stoma, depending on the site and number of perforations, severity of peritonitis, and general condition of the patient. Thereafter, the patient is managed with antibiotics and continued postoperative care. Ileostomy serves the purpose of diversion, decompression, and exteriorization. Primary ileostomy has been found to be superior to other surgical procedures as far as the morbidity and mortality are concerned and especially so in moribund patients presenting late in course of their illness, where it proves to be a lifesaving procedure [6]. These are the types of patients that usually come to our surgical emergencies in India.

Though ileostomy is a lifesaving procedure in such cases, it may result in significant number of complications as well. A small intestinal diverting stoma carries significant morbidity, mostly due to fluid/electrolyte imbalance and nutritional depletion. Peristomal skin irritation is perhaps the commonest complication of ileostomy leading to skin excoriation [7]. Other complications after ileostomy are bleeding, ischemia, obstruction, prolapse, retraction, stenosis, para-stomal herniation, fistula formation, residual abscess, wound infection, and incisional hernia. In addition, ileostomy is known to adversely affect the quality of life due to physical restrictions and psychological problems [8].

The present study is aimed to analyze the epidemiology and presentation of such cases undergoing ileostomy for perforation peritonitis.

## 2. Material and Methods

The present study was a prospective observational study conducted in the Department of Surgery, Postgraduate Institute of Medical Sciences, Rohtak, a tertiary care center in North India. The study was conducted over a period of two and a half years (August 2011 to December 2013) after getting approval from the institutional ethical committee. Forty-one patients admitted with perforation peritonitis due to ileal perforation and undergoing emergency laparotomy with ileostomy were included in the study. Those cases of ileal perforation managed by primary closure or small gut resection and anastomosis were excluded from the study. The criteria used for deciding the type of operative procedure are given in Table 1.

All patients were thoroughly evaluated with detailed history, clinical examination, and blood investigations including complete blood counts, blood urea, X-ray chest in erect position, Widal test, and blood culture. The procedure was explained to the patients and written consent was taken regarding the stoma formation. All the cases were managed with intravenous fluids for resuscitation, nasogastric tube for gut decompression, urethral catheterization for monitoring urine output, third generation cephalosporins, and analgesics. After initial resuscitation in emergency department, patients underwent emergency laparotomy through midline incision. The intraoperative findings, namely, site, number, and size of perforations, extent of peritonitis, condition of gut, status of lymph nodes, and mesentery, were recorded and thorough peritoneal lavage was done. End or loop ileostomy was created as per the standard methods. Patients were monitored postoperatively and their histopathology reports were compiled.

## 3. Results

A total of 41 patients suffering from generalized peritonitis due to ileal perforation and managed with ileostomy were included in the study and their demographic and clinical profile was analyzed (Table 2). The majority of these cases belonged to the age group of 21–30 years with 34 (82.9%) being males. Moreover, 80% of the patients were from rural background. At the time of presentation, the patients had pain abdomen (100%), vomiting (92.7%), fever (85.3%), and obstipation (73%). On examination, there was abdominal tenderness (100%), guarding (95.1%), absent bowel sounds (85.4%), and abdominal distension (70.7%).

Mean body mass index (BMI) was 19.6 ± 1.66 with a range of 15.4–25.3. Only one patient (2.4%) was moderately obese, whereas 22% cases were underweight. Comorbidities were recorded in all the cases with chest infection being the commonest (22%) followed by heart diseases (9.7%), diabetes (4.8%), and hypertension (2.4%). Most of the patients were chronic smokers. Among other comorbidities, one patient had hypothyroidism, carcinoma base of tongue (after chemoradiation), and hepatitis B.

Blood investigations showed that 51.2% patients had total leucocyte counts more than 11,000/mm^3^ whereas only one patient had counts less than 4000/mm^3^. Preoperative blood urea was raised in majority of the patients. Widal test was positive in 36.6% patients while only one patient had a positive blood culture, with the isolate being* Citrobacter*.

On chest X-ray (erect film), thirty-seven patients (90.2%) had air under diaphragm suggestive of gut perforation. Four patients had associated pleural effusion and two had changes suggestive of pneumonitis. Four patients had normal skiagram. In view of clinical suspicion of perforation peritonitis in these four cases, contrast enhanced computerized tomography (CECT) scan of the abdomen was done that confirmed the diagnosis. All the patients were hemodynamically unstable and required resuscitation before surgery with intravenous fluids and inotropes. Resuscitation period ranged from 4 to 72 hours with a mean duration of 14.7 hours. The majority of the patients required preoperative resuscitation for 7–12 hours.

On exploration, all the patients had generalized peritonitis and diffuse enteritis with multiple perforations in the distal ileum (100%). Mesenteric edema and thickening were seen in more than half the patients (65.9%) whereas almost a quarter of patients had mesenteric lymphadenopathy (27.8%). Histopathological examination of resected gut specimen revealed nonspecific inflammation (56%), typhoid perforation (24.4%), and tubercular inflammation (19.5%) (Table 2).

In postoperative period, various complications seen were stomal discoloration (14.6%), peristomal skin excoriation (41.4%), wound sepsis (24.3%), intra-abdominal abscess (17%), and burst abdomen (4.8%). Three patients had postoperative septicemia and expired (7.3%).

## 4. Discussion

Peritonitis due to hollow viscus perforation is commonly encountered in surgical practice. It is caused by the introduction of infection into the otherwise sterile peritoneal environment through perforation of bowel. The spectrum of aetiology of perforation in tropical countries continues to be different from its Western counterpart. In contrast to Western countries where lower gastrointestinal tract perforations predominate, upper gastrointestinal tract perforations constitute the majority of cases in India [1]. Spontaneous ileal perforation remains a formidable surgical condition in developing countries. Typhoid fever is the predominant cause of nontraumatic ileal perforation while other causes include tuberculosis, nonspecific inflammation, obstruction, radiation enteritis, and Crohn's disease.

Though surgery is accepted as the definite treatment, the choice of exact surgical procedure remains controversial. Most series report simple closure of the perforation or resection and anastomosis as choice of procedure. These procedures though appealing are not free of complications especially in an emergency setup. Of all the postoperative complications reported, faecal fistula remains the most life-threatening; the rate of its occurrence has been reported to be around 12% with a very high mortality rate [14]. In view of this alarming situation, a shift in favour of a defunctioning protective ileostomy has been observed in recent years. Ileostomy is a lifesaving procedure, particularly in those cases where there are fulminant enteritis and generalized peritonitis of long duration. Various criteria used for deciding the type of operative procedure can be based on preoperative conditions and intraoperative findings in such cases (Table 1).

In most of the studies from Asia, mean age of the patients presenting with ileal perforation is around 35 to 40 years and the findings in the present study were the same [1, 15–17]. According to Mock et al., morbidity and mortality increase as age advances possibly due to comorbidities and poor immunity [18]. Park et al. [19] also had similar observations but only for early complications; late complications did not correlate with age.

The incidence of perforation peritonitis due to ileal perforation is significantly more in male population as seen in the present and the previous similar studies. In most of the studies, male patients contributed more than 75% of total cases [1, 15–17]. This is possibly because males indulge in outdoor activities and are more prone to GI infections and its attendant complications including perforation peritonitis. However, Park et al. found that there was no relation between sex and complications in these cases [19]. The majority of the patients in the present study and previous similar studies presented with pain abdomen, vomiting, constipation, and fever. Fever is a common symptom in cases of typhoid perforation peritonitis and ileal perforation is usually seen in the third week of illness. Shock and dehydration were seen more in our patients compared to other studies, indicating that patients in the present study were sicker and underwent ileostomy as a lifesaving measure. The clinically stable cases underwent primary closure/resection anastomosis of small gut and were excluded from the present study.

Associated comorbid illnesses seen in Western population included cardiac diseases and diabetes mellitus whereas, in our study, most of the patients had poor chest condition as most the common comorbidity, probably because of smoking habits and associated chronic obstructive pulmonary disease (COPD). Patients of COPD are more prone to postoperative complications like pneumonitis, poor healing, and wound dehiscence.

Compared to our study, the patients in previous studies had a normal BMI or were obese. Chun et al. [12] encountered more than 65% patients with BMI >25. They found that obesity was a significant risk factor for overall ileostomy complications, outpatient complications, and severe peristomal skin problems that required additional care. Moreover, in their study, patients with a BMI >30 had the highest number of ileostomy related complications. Leong et al. [20] suggested that, in obese patients, an end ileostomy may be the only option that provides sufficient length for the stoma to extend through the abdominal wall without tension because of the short thickened fatty mesentery. Park et al. [19] found that there is no significant relation in BMI and early or late complications. Faunø et al. [13] found a weak association between high BMI and parastomal hernia. Most of the patients in our study were poorly nourished and had BMI ranging from 15.4 to 25.3, with a mean of 19.6 ± 1.66. In our study, complications like parastomal skin excoriation, wound sepsis, stomal retraction, and prolapse had no significant correlation with BMI (Table 3).

Most patients with ileal perforation peritonitis have one or two perforations. Sometimes, there may be multiple perforations especially in immune-compromised patients [21]. Mock et al. [18] in their series of 221 patients found that the increased number of perforations was associated with a significantly higher mortality rate. In the present study, all the patients had multiple perforations with severe enteritis and postoperative mortality occurred in three cases (7.3%).

## 5. Conclusion

Temporary defunctioning protective ileostomy in moribund cases of peritonitis due to ileal perforation is a lifesaving procedure. Apart from reducing mortality, it plays a vital role in decreasing the incidence of complications like faecal fistula. While some advocate primary repair or anastomosis as methods of source control in these cases, ileostomy may be a more prudent alternative in an Indian setting where most of the patients have low BMI and usually present late with severe sepsis and generalized peritonitis. It is essential that an emergency surgeon be well versed in all the techniques of source control in such cases and choose the appropriate source control measure.

## Figures and Tables

**Table 1:** Criteria for deciding the type of operative procedure.

Operative procedure	Criteria
Primary closure	Patient presenting within 24 hrs of perforation, being hemodynamically stable, having minimal or no resuscitation required preoperatively, localized peritonitis, mild enteritis, single perforation, and no other areas of impending perforation

Resection and anastomosis	Patient presenting within 24 hrs of perforation, being hemodynamically stable, having minimal or no resuscitation required preoperatively, localized peritonitis, mild to moderate enteritis, multiple perforations, and areas of impending perforation

Ileostomy	Patient presenting > 24 hrs after perforation, being hemodynamically unstable, having resuscitation required preoperatively, generalized peritonitis, severe enteritis, multiple perforations, and areas of impending perforation

**Table 2:** Demographic and clinical profiles of patients.

Mean age (years)	38.31 ± 18.99
Sex (male/female)	34/7
Pain *n* (%)	41 (100)
Vomiting *n* (%)	37 (92)
Constipation *n* (%)	30 (73)
Shock *n* (%)	41 (100)
Fever *n* (%)	35 (85.3)
Dehydration *n* (%)	32 (78.1)
Distension *n* (%)	29 (70.7)
Abdominal tenderness *n* (%)	41 (100)
Abdominal guarding *n* (%)	38 (95.1)
Average time of resuscitation (hours)	14.7
Body mass index *n* (BMI)	19.6 ± 1.66

**Table 3:** Mean BMI of patients in various studies.

Study (number of patients)	Mean BMI
Arumugam et al., 2003 [9] (*n* = 97)^*∗*^	24.5 ± 4.66
El-Hussuna et al., 2012 [10] (*n* = 159)^*∗*^	27 ± 5.12
Sharma et al., 2013 [11] (*n* = 5401)	25.5
Chun et al., 2012 [12] (*n* = 123)^*∗*^	29.6
Faunø et al., 2012 [13] (*n* = 700)^*∗*^	28 ± 5.32
Our study (*n* = 41)	19.6 ± 1.66

(^*∗*^Ileostomy done electively for colorectal cancer, polyposis coli, and inflammatory bowel disease.)
